# How international are the editorial boards in the field of hand research? A cross-sectional study of leading subspecialty hand journals

**DOI:** 10.1186/s13018-023-04068-x

**Published:** 2023-08-07

**Authors:** Tianlin Wen, Donghua Liu, Xingxuan Li, Yan Zhang, Zhiwei Jia, Yaohong Wu, Wei Li

**Affiliations:** 1 Department of Orthopedics, Dongzhimen Hospital, Beijing University of Chinese Medicine, Beijing, China; 2 Department of TCM Orthopedics, Sixth Medical Center of PLA General Hospital, Beijing, China; 3 Department of Spine Surgery, Ganzhou People’s Hospital, Ganzhou, China; 4 Department of Sports Medicine, Fourth Medical Center of PLA General Hospital, Beijing, China

**Keywords:** Editorial board members, Hand, Journal, Publication

## Abstract

**Background:**

Although low- and middle-income countries (LMIC) have great disease burden, but the lack of studies from LMIC have been shown in several fields. Multiple researchers from LMIC perceive editorial bias against their studies. Editorial board members (EBMs) from LMIC are under-represented across many medical journals. It is still unclear whether this phenomenon exists in the field of hand research. The purpose of this study was to investigate the composition of EBMs in leading subspecialty hand journals, and to reveal the international representation of EBMs in the field of hand research.

**Methods:**

This cross-sectional study included seven leading subspecialty hand journals. The EBMs were obtained from the journals’ websites. The country affiliations of EBMs were categorized based on their locations and economy status. The composition of EBMs was investigated.

**Results:**

There were 211 EBMs in the seven journals. A total of 185 EBMs (87.7%) were affiliated with high-income countries (HIC), 18 (8.5%) with upper middle-income countries, and 8 (3.8%) with lower middle-income countries. None EBMs were affiliated with low income countries. The EBMs were affiliated with 30 countries. The biggest number of EBMs were affiliated with the USA 74 (35.07%), followed by the United Kingdom (45, 21.33%), and France (13, 6.16%). Most of EBMs were based in Europe and Central Asia (86, 40.8%) and North America (81, 38.4%).

**Conclusions:**

The EBMs of leading subspecialty hand journals are dominated by HIC with a very low representation of LMIC. There is a need to make the editorial boards more international in the field of hand research.

## Background

Low- and middle-income countries (LMIC) have more than 80% of the global population [1]. Most of global disease burden lies in LMIC due to the great number of patients [2]. The burden of hand disease in LMIC is immense and increasing [3–5]. Although hand problems are typically not life-threatening, they give rise to substantial morbidity [4]. Patients afflicted with hand diseases often face difficulties in resuming their work activities, lose their capacity for self-care, and suffer a general decline in their overall quality of life [4, 6]. Hand surgeons are presented with a unique opportunity to offer life-altering care to individuals afflicted with hand disease [7]. However, the limited resources in LMIC are an obvious challenge in the management of hand conditions and disabilities [8].

In recent years, great progress has been found in the treatment of hand disease because of the global contributions [9–12]. Publications is very important to the research progress [1, 13–17]. It has been reported that there is a significant increase in the global hand-related research [18]. However, investigators from LMIC only contribute 7.97% of publications in subspecialty hand journals [18]. In the field of hand surgery, it is observed that surgeons from high-income countries (HIC) often prioritize the treatment of degenerative diseases [6, 19]. On the other hand, hand trauma is a prevalent occurrence in LMIC and is primarily attributed to factors such as traffic accidents, occupational hazards, incidents of violence, and burns [4, 6]. Many researchers affiliated with LMIC feel that it is very difficult for them to publish their papers in high impact journals [13, 20]. Some studies show that editorial bias may be an important factor for low percentage of papers affiliated with LMIC [14, 15, 21–26]. The composition of editorial board members (EBMs) as an important indicator is widely used to evaluate the international representation of subspecialty journals [21–24]. EBMs from LMIC are under-represented in some medical journals [15, 21–30]. Nevertheless, it still unclear whether this phenomenon exists in hand journals. The purpose of this study was to investigate the composition of EBMs in leading subspecialty hand journals, and to reveal the international representation of EBMs in the field of hand research.

## Methods

This is a cross-sectional study of subspecialty hand journals. This study did not involve human and animals, so approval of Institutional Reviewed Board was not required. On July 19, 2023, the Journal Citation Reports for the year 2022 were used to identify the high-impact subspecialty hand journals. Seven leading subspecialty hand journals with impact factors were included, including *Journal of Hand Therapy* (JHT), *Journal of Hand Surgery (American Volume)* (JHSA), *Journal of Hand Surgery (European Volume)* (JHSE), *Hand Clinics* (HC), *Hand Surgery & Rehabilitation* (HSR), *Hand Therapy* (HT), and *Journal of Hand Surgery (Asian-Pacific Volume)* (Table 1).

**Table 1:** The information of leading subspecialty hand journals

Journal	Abbreviation	Country of publication	Impact factor
Journal of hand therapy	JHT	USA	2.0
Journal of hand surgery (American volume)	JHSA	USA	1.9
Journal of hand surgery (European volume)	JHSE	England	1.8
Hand clinics	HC	United States	1.1
Hand surgery and rehabilitation	HSR	France	1.1
Hand therapy	HT	England	1.0
Journal of hand surgery (Asian-Pacific volume)	JHSAP	Singapore	0.5

The EBMs were obtained from the journals’ websites. The number of EBMs and their affiliated countries were collected. The country affiliations of EBMs were categorized by their locations and economy status based on the World Bank (www.worldbank.org). The locations included North America (NA), Europe and Central Asia (ECA), East Asia and Pacific (EAP), Latin America and Caribbean (LAC), Middle East and North Africa (MENA), South Asia (SA), and Sub-Saharan Africa (SSA). The economic status included the following income groups: HIC, upper middle-income countries (UMIC), lower middle-income countries, and low income countries.

The countries with 1% or more of the total EBMs were defined as the major countries [18, 31–34]. The population and gross domestic product (GDP) were used to normalize the number of EBMs. The World Bank was reviewed to collect the data of population and GDP.

This study was to analyze the characteristics of EBMs, not to test hypotheses with the relative importance of EBMs of the countries. Therefore, descriptive statistics (e.g., total and percentage) were calculated.

## Results

**Fig. 1:**
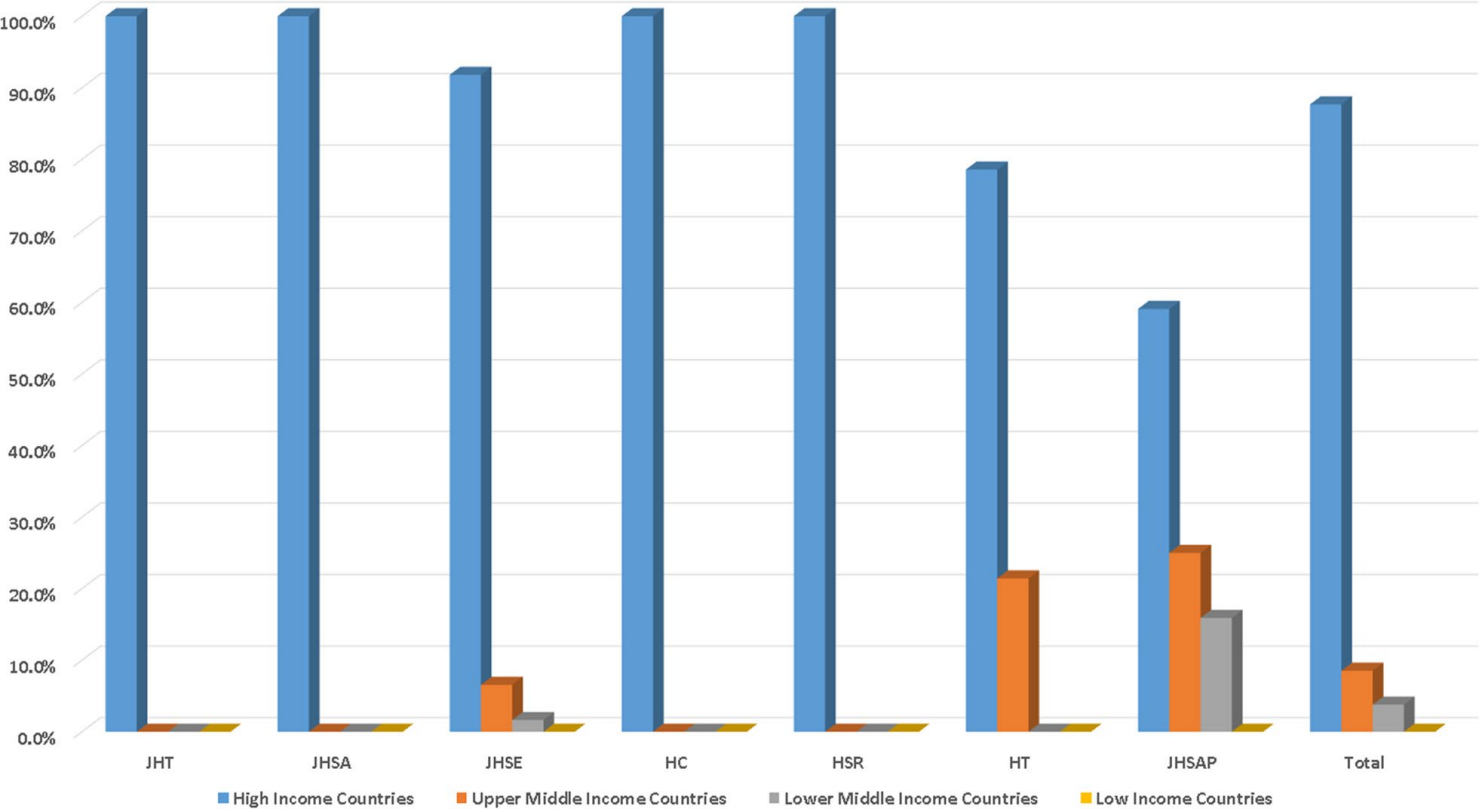
EBMs classified by income groups

There were 211 EBMs in the seven subspecialty hand journals. A total of 185 EBMs (87.7%) were affiliated with HIC, 18 (8.5%) with UMIC, and 8 (3.8%) with lower middle-income countries. None EBMs were affiliated with low income countries. All the EBMs of JHT, JHSA, HC, and HSR were affiliated with HIC (100%). The EBMs of JHSE were affiliated with HIC (56, 91.8%), UMIC (4, 6.6%), and lower middle-income countries (1, 1.6%). The EBMs of HT were affiliated with HIC (11, 78.6%) and UMIC (3, 21.4%). The EBMs of JHSAP were affiliated with HIC (26, 59.1%), UMIC (11, 25.0%), and lower middle-income countries (7, 15.9%). The income groups are illustrated in Fig. 1.

**Fig. 2:**
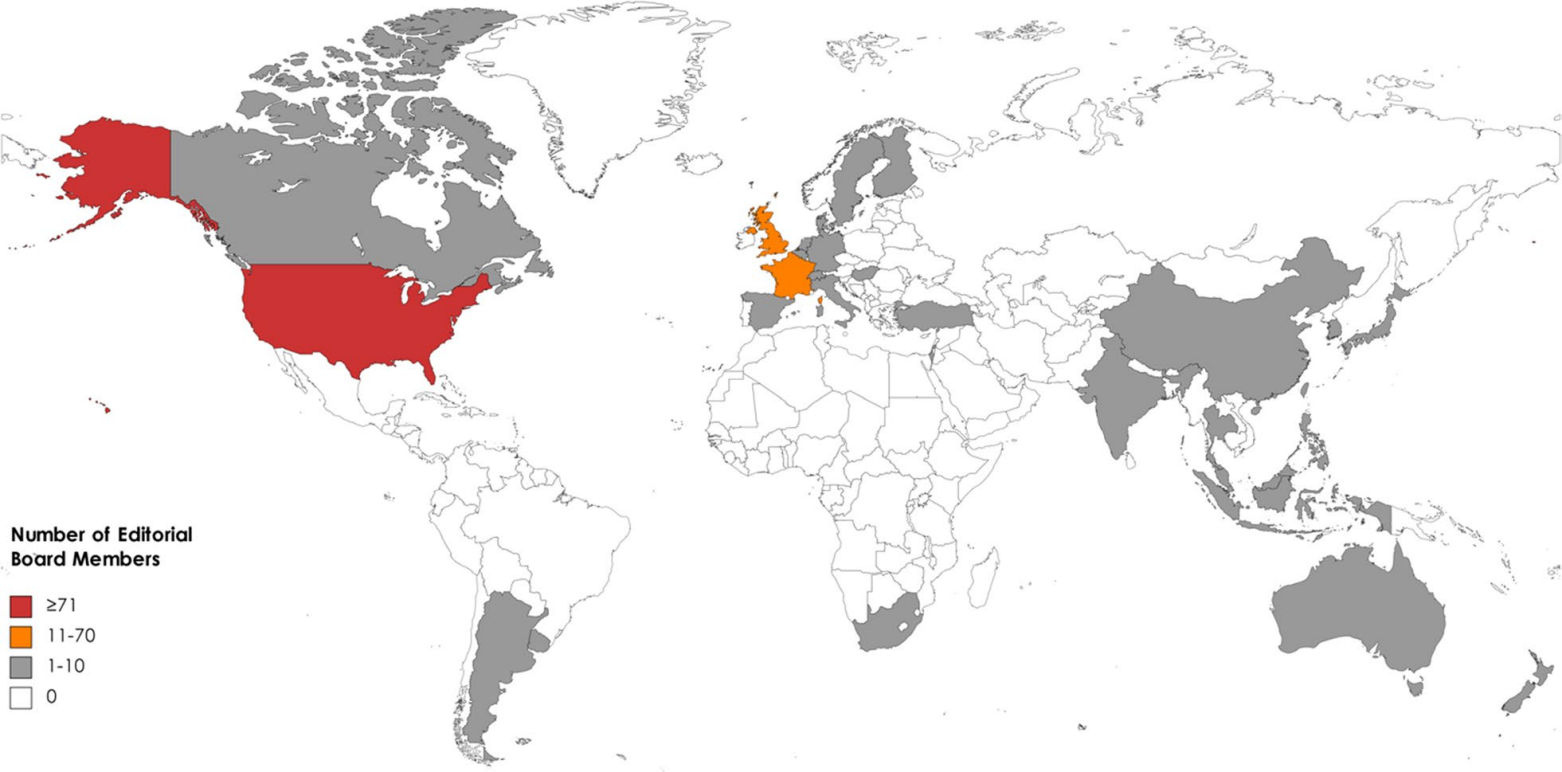
The worldwide distributions of EBMs

The EBMs were affiliated with 30 countries. There were 21 HIC, 7 UMIC, and 2 lower middle-income countries. The biggest number of EBMs were affiliated with the USA 74 (35.07%), followed by the United Kingdom (45, 21.33%), and France (13, 6.16%). The global distributions of EBMs are illustrated in Fig. 2.

**Fig. 3:**
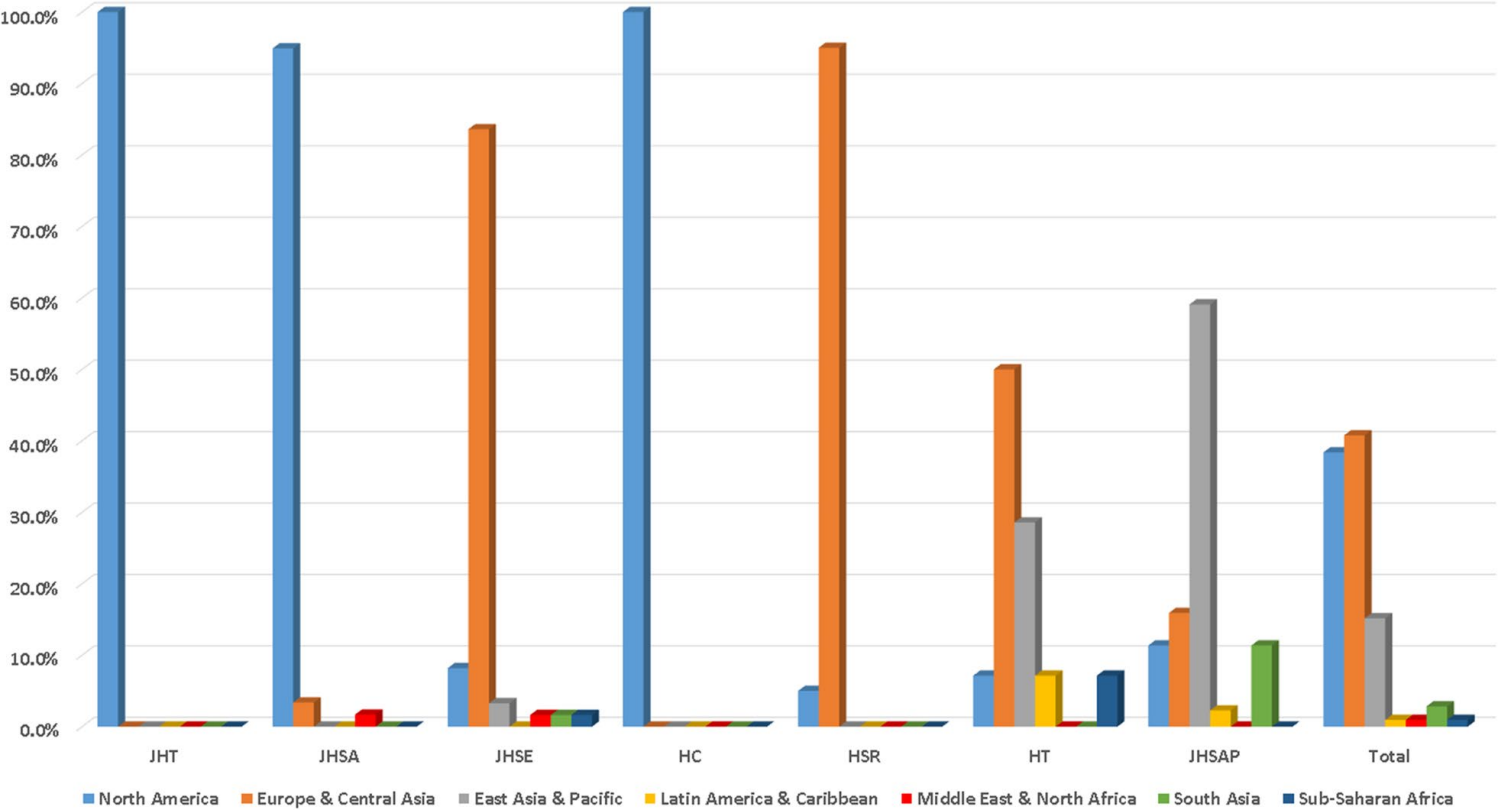
EBMs classified by regions

The seven journals were from four countries, including the USA. England, France, and Singapore (Table 1). JHSA, JHT, and HC were from the USA. JHSE and HT were from England. HSR was from France. JHSAP was from Singapore. Most of the EBMs were based in Europe and Central Asia (86, 40.8%) and North America (81, 38.4%), followed by EAP (15.2%), SA (2.8%), MENA (0.9%), SSA (0.9%), and LAC (0.9%). The EBMs of both JHT and HC were only affiliated with NA (100%). The EBMs of JHSA were affiliated with three regions, including NA (94.9%), ECA (3.4%), and MENA (1.7%). The EBMs of JHSE were affiliated with six regions, including ECA (83.6%), NA (8.2%), EAP (3.3%), MENA (1.6%), SA (1.6%), and SSA (1.6%). The EBMs of HSR were affiliated with two regions, including ECA (95%) and NA (5%). The EBMs of HT were affiliated with five regions, including ECA (50%), EAP (28.6%), NA (7.1%), LAC (7.1%), and SSA (7.1%). The EBMs of JHSAP were affiliated with five regions, including EAP (59.1%), ECA (15.9%), NA (11.4%), SA (11.4%), and LAC (2.3%). The geological compositions of EBMs are illustrated in Fig. 3.

The characteristics of 15 major countries are listed in Table 2. Six of these countries were affiliated with ECA, 6 with EAP, 2 with NA, and 1with SA. Twelve of them were HIC, 2 was UMIC, and 1 was lower middle-income country. When the quantity of EBMs was normalized by the population, Singapore had the highest research output (91.7), followed by Switzerland (69.0), and the United Kingdom (66.8). When considering the GDP, the United Kingdom (14.1) had the best record, followed by Singapore (12.6), and Switzerland (7.4).

**Table 2:** The major countries of EBMs of leading subspecialty hand journals

Countries	Region	Income group	No. of EBMs	Proportion (%)	No. per 100 million populations	No. per $ 1000 billion GDP
USA	NA	HIC	74	35.07	22.3	3.2
United Kingdom	ECA	HIC	45	21.33	66.8	14.1
France	ECA	HIC	13	6.16	19.3	4.4
China	EAP	UMIC	8	3.79	0.6	0.5
Canada	NA	HIC	7	3.32	18.3	3.5
Switzerland	ECA	HIC	6	2.84	69.0	7.4
India	SA	LMC	6	2.84	0.4	1.9
The Netherlands	ECA	HIC	5	2.37	28.5	4.9
Singapore	EAP	HIC	5	2.37	91.7	12.6
South Korea	EAP	HIC	4	1.90	7.7	2.2
Japan	EAP	HIC	4	1.90	3.2	0.8
Australia	EAP	HIC	4	1.90	15.5	2.6
Belgium	ECA	HIC	3	1.42	25.9	5.0
Spain	ECA	HIC	3	1.42	6.3	2.1
Thailand	EAP	UMIC	3	1.42	4.3	5.9

*GDP* gross domestic product, *NA* North America, *EAP* East Asia and Pacific, *MENA* Middle East and North Africa, *ECA* Europe and Central Asia, *SA* South Asia, *HIC* high income countries, *UMIC* upper middle-income countries, *LMC* lower middle-income countries

**Fig.4:**
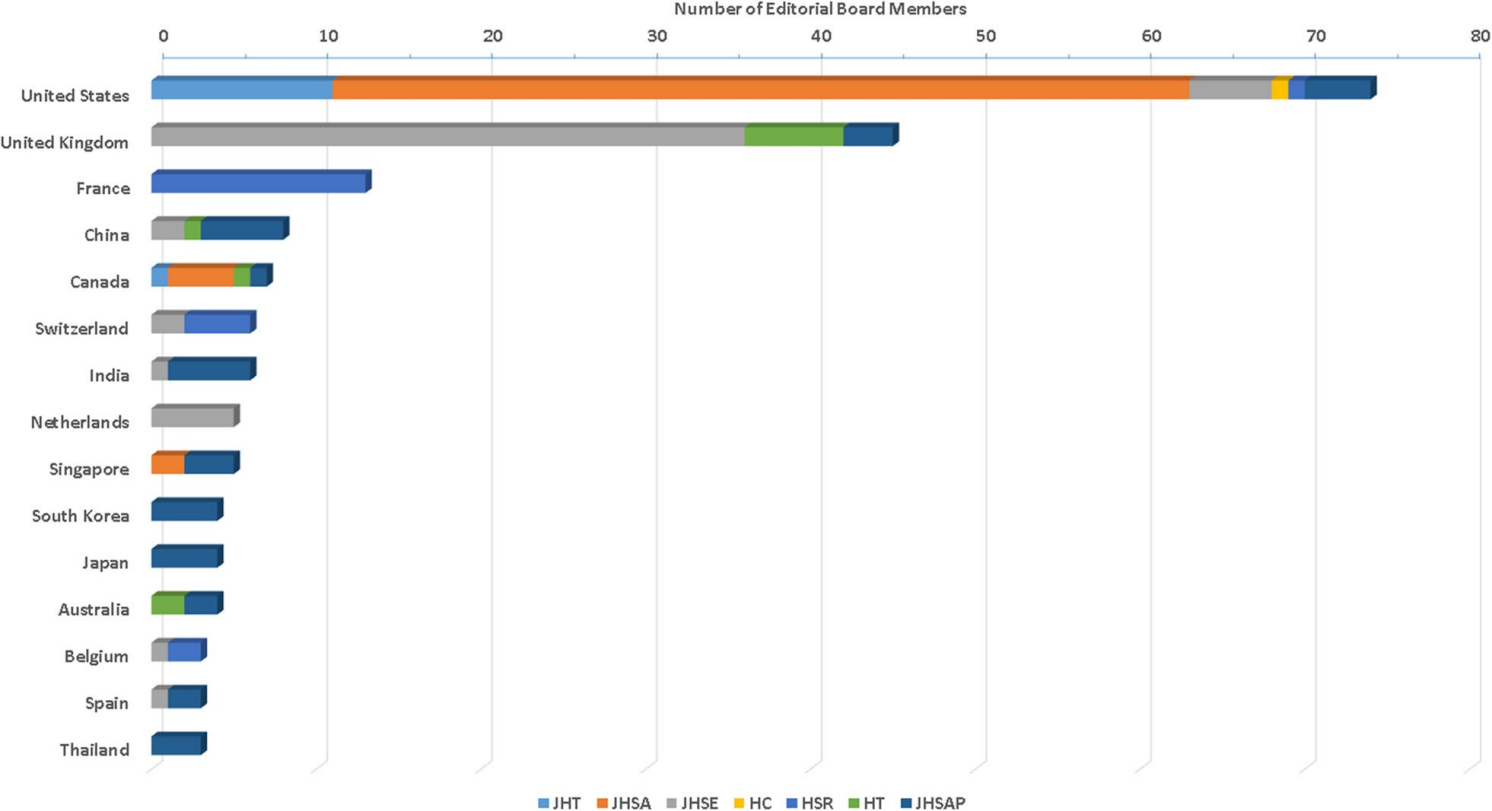
The journal distributions of EBMs from the major countries

The largest number of EBMs from the USA were in JHSA. The largest number of EBMs from the United Kingdom and The Netherlands were both in JHSE. The largest number of EBMs from France were in HSR. The largest number of EBMs from India were in JHSAP. The journal compositions of EBMs of the major countries are illustrated in Fig. 4.

## Discussion

The development of hand research could be attributed to worldwide scientific efforts [18]. The publications sharing new knowledge are vital in scientific activities [11, 12, 35, 36]. The preferred publications of journals are determined by the EBMs [27, 28, 37, 38]. Significantly less publications from LMIC have been found in some fields [15, 29, 30, 39]. Although this phenomenon could be ascribed to multiple factors, editorial bias catches more and more attentions from the researchers affiliated with LMIC [14, 23–26, 29]. Some authors from LMIC perceived that they were unequally treated by EBMs of the journals [29, 37, 38, 40]. Low representation of EBMs from LMIC have been found in some medical fields [15, 21, 25, 26, 29, 30, 39]. As far as we know, it has not been determined in hand journals.

The four leading countries included the United States, the United Kingdom, France, and China. These countries had nearly 70% of total EBMs. This finding indicates that EBMs centered in a few countries. Importantly, the top two countries, namely the United States and the United Kingdom, possessed more than 50% of the total EBMs. It is worth noting that both of these countries are English-speaking nations. The cultural dominance of English-speaking countries may significantly shape the characteristics and content of hand journals [14, 15, 23, 24, 41]. Therefore, EBMs from the under-represented countries may play a limited role in policies of the journals [23, 25, 42].

In total, nearly 80% of total EBMs were affiliated with NA and ECA, indicating that EBMs were not with wide distribution. It could be attributed to the fact that the three leading countries are based in NA and ECA. Most of EBMs were affiliated with these countries. There is a need to recognize the imbalance distributions of EBMs by the scientific community of the hand research [14, 15, 23–28, 42].

This study found that nearly 90% of EBMs of leading hand journals were affiliated with HIC, and limited EBMs with LMIC. Hence, under-representation of EBMs from LMIC is revealed in hand journals, which is similar with the findings in other medical fields [15, 25, 26, 29, 37, 40]. The LMIC have larger populations and patients comparing with HIC, but the percentage of EBMs is extremely low in hand journals. This may play a negative role to the submission from LMIC [24, 37, 38]. Active involvement of local researchers throughout the research process is essential as they often have a deeper understanding of the local context and needs [43]. Additionally, they are better positioned to advocate for implementing the research findings [44]. However, conducting research in LMIC differs significantly from HIC [45, 46]. Many clinicians in LMIC do not have dedicated time for research due to heavy clinical workloads [45]. Furthermore, there are barriers that restrict access to current scientific literature for authors and readers in LMIC [41, 46]. To improve access to scientific literature in LMIC, open access is a potential solution [46]. There is increasing evidence supporting the various benefits of open access publication, including economic, social, and academic advantages [46, 47]. However, publishing in open access journals can be costly [41, 46, 47]. Authors from LMIC often have to allocate a larger portion of their available income to publish open access publication [45, 46]. These factors may result in limited contributions to the overall research output by researchers from LMIC. Consequently, the scarcity of publications from LMIC researchers can lead to reduced research activity focused on diseases predominantly affecting LMIC populations [14, 23, 24, 37, 38, 40]. It is imperative to undertake efforts aimed at strengthening health research capacity in LMIC and addressing the inadequate representation of LMIC researchers in the editorial boards of hand journals [43, 44]. The present study also revealed that when considering the membership of EBMs from LMIC standardized by their large populations, the relative percentage of LMIC representation appears significantly smaller [23, 24]. To address this issue, alternative methods of normalization could be explored. Instead of utilizing population size as a normalization factor, it may be more sensible to normalize by the number of researchers specializing in hand research within each country. However, obtaining such data poses challenges in the field of hand research, as only a limited number of researchers are engaged in full-time research [21, 22, 48]. Nevertheless, this finding serves as an indication of the overall lower percentage of EBMs from LMIC in comparison to other regions [21, 22, 45].

A total of three journals, including JHSA, JHT, and HC, are affiliated with the USA. Interestingly, the highest percentages of EBMs of JHSA, JHT, and HC are affiliated with the USA. Similarly, three journals including JHSE, HSR, and HT are from the Europe. The highest percentages of EBMs of JHSE, HSR, and HT are affiliated with the Europe. JHSAP are from Singapore. The highest percentages of EBMs of JHSAP are affiliated with the Asia. Although these are international journals in the field of hand research, these results indicate that there is a tendency to appoint EBMs from their locations in the leading hand journals, which should be noted by the hand journals [21–25].

The objective of this study was to analyze the composition of EBMs of hand journals. The findings of this study indicate a notable under-representation of EBMs from LMIC in leading hand journals. While it is evident that there is a low percentage of EBMs from LMIC, it remains uncertain whether this indicates an editorial bias in hand journals. The inclusion of a diverse range of EBMs can play a crucial role in ensuring diverse and balanced perspectives in research publications [23, 24, 37, 38]. It is important to recognize that the imbalanced composition of EBMs may contribute to an inherent bias [23, 26, 37, 38, 40], potentially leading to an increased focus on diseases prevalent in HIC and a decrease in articles addressing healthcare in LMIC [1, 23, 24, 31]. The low representation of LMIC in hand journals should be acknowledged by both the journals themselves and the scientific community involved in hand research. It is the responsibility of leading hand journals to actively work toward minimizing or eliminating potential biases by fostering a well-balanced representation of EBMs from various regions and income countries [23, 24, 31, 38]. Future measures may include appointing more EBMs from LMIC and implementing rotation systems among EBMs from different countries. [15, 25, 29, 38].

Although this study provides evidence of the under-representation of EBMs from LMIC in leading hand journals, it is important to consider several factors regarding this issue. First, one possible explanation for the low percentage of EBMs from LMIC is the limited pool of qualified editors available for appointment in subspecialty hand journals. [1, 22, 29, 31, 39, 42]. These leading journals are primarily published in English, and therefore, EBMs must possess proficiency in English to effectively communicate with other editors and authors. However, it should be noted that a significant number of LMIC are non-English speaking countries. Additionally, researchers from LMIC may lack extensive experience in high-level research, which may hinder their ability to contribute effectively as editors for leading journals [1, 29, 39, 42]. Second, although these leading journals strive for a global perspective, they primarily cater to local needs, problems, and readership [1, 20, 29, 42]. Consequently, some editorial bias may be inevitable. If EBMs from HIC devote more attention to assisting authors from LMIC in presenting and formatting their research papers, it can be viewed as a positive development for hand journals. Third, increasing the representation of EBMs from LMIC is not the sole solution for addressing the under-representation issue. Each hand journal requires an editor who is willing to invest additional time and effort in managing submissions from LMIC.

There are several limitations in the current study that should be acknowledged. First, it is important to note that the selected leading hand journals included in this study are predominantly published in English. Therefore, it is inevitable that language bias may exist as a potential limitation [15, 23–26, 38]. Second, the sample size of this study is limited as it includes only seven subspecialty hand journals. Nevertheless, it is worth noting that these journals are considered to be leading publications in the field and are likely to be representative of major international hand journals. [14, 15, 25, 26].

## Conclusions

The EBMs of leading subspecialty hand journals are dominated by HIC with a very low representation of LMIC. There is a need to make the editorial boards more international in the field of hand research.

## Data Availability

The data generated in this study are available from the corresponding author on reasonable request.
